# How Donor and Surgical Factors Affect the Viability and Functionality of Human Hepatocytes Isolated From Liver Resections

**DOI:** 10.3389/fmed.2022.875147

**Published:** 2022-05-11

**Authors:** Estela Solanas, Nieves Sanchez-Fuentes, Alejandro Serrablo, Alberto Lue, Sara Lorente, Luis Cortés, Angel Lanas, Pedro M. Baptista, M. Trinidad Serrano

**Affiliations:** ^1^Aragón Institute for Health Research (IIS Aragón), Zaragoza, Spain; ^2^Centro de Investigación Biomédica en Red Enfermedades Hepáticas y Digestivas (CIBERehd), Madrid, Spain; ^3^Hepato-Pancreato-Biliary Surgical Division, Miguel Servet University Hospital, Zaragoza, Spain; ^4^Department of Digestive Diseases, University Clinic Hospital Lozano Blesa, Zaragoza, Spain; ^5^ARAID Foundation, Zaragoza, Spain; ^6^Department of Biomedical Engineering, Carlos III University of Madrid, Madrid, Spain

**Keywords:** humans, liver, hepatocytes, hepatectomy, cell separation, warm ischemia

## Abstract

Liver resections are a significant source of primary human hepatocytes used mainly in artificial liver devices and pharmacological and biomedical studies. However, it is not well known how patient-donor and surgery-dependent factors influence isolated hepatocytes’ yield, viability, and function. Hence, we aimed to analyze the impact of all these elements on the outcome of human hepatocyte isolation.

**Patients and methods:**

Hepatocytes were isolated from liver tissue from patients undergoing partial hepatectomy using a two-step collagenase method. Hepatocyte viability, cell yield, adhesion, and functionality were measured. In addition, clinical and analytical patient variables were collected and the use or absence of vascular clamping and its type (continuous or intermittent) plus the ischemia times during surgery.

**Results:**

Malignant disease, previous chemotherapy, and male gender were associated with lower hepatocyte viability and isolation cell yields. The previous increase in transaminases was also associated with lower yields on isolation and lower albumin production. Furthermore, ischemia secondary to vascular clamping during surgery was inversely correlated with the isolated hepatocyte viability. An ischemia time higher than 15 min was related to adverse effects on viability.

**Conclusion:**

Several factors correlated with the patient and the surgery directly influence the success of human hepatocyte isolation from patients undergoing liver resection.

## Introduction

Human hepatocytes are the basis for vital biomedical applications, such as bioartificial liver devices, used as bridge therapy in acute and acute-on-chronic liver failure, and *in vitro* models used for countless biomedical, pharmacological, and toxicological studies. In addition, these cells are still considered the gold standard for evaluating *in vitro* metabolism and hepatotoxicity during the development of novel drugs and chemical compounds.

Likewise, human hepatocyte transplantation has been postulated as an alternative to orthotopic liver transplantation in certain liver disorders, especially those of a metabolic nature (1, 2). It has also been used as functional support until the procurement of a healthy liver for transplantation or the regeneration of a patient’s damaged liver after fulminant failure (1, 3). However, in any of its applications, hepatocyte transplantation requires the infusion of a large number of fully functional human hepatocytes.

Therefore, undoubtedly, both for hepatocyte transplantation and for the progress and development of bioartificial liver devices and *in vitro* models for pharmacological and toxicological studies, it is necessary and critical to have sufficient fully functional primary human hepatocytes.

Primary human hepatocytes are usually obtained by cell isolation using a two-step collagenase perfusion technique from freshly excised livers not used for orthotopic transplantation or from liver pieces obtained during partial hepatectomy (4–6). The shortage of organs for transplantation and the progress in orthotopic transplantation of, until recently, discarded livers due to their low quality significantly reduced the number of rejected livers for transplantation used for hepatocyte isolation, making liver resections one of the primary sources for isolating these cells. However, the quantity and quality of the hepatocytes obtained by this procedure will depend not only on the isolation technique itself but also on other factors, such as the donor’s characteristics, the surgical process itself, or the preservation of the tissue until it is processed.

Among factors derived from the surgical procedure or from preserving the resected tissue until isolation, ischemia-reperfusion is one of the most studied fields in literature. Although adverse effects of ischemia times, both warm (*in vivo*, during surgery for vascular occlusion) and cold (*ex vivo*, the time from tissue resection until cell isolation), suffered by the hepatic tissue on the number and viability of the isolated hepatocytes have been demonstrated, results of the different studies in this topic have been quite diverse and even contradictory. Thus, concerning the effect of warm ischemia, Alexandre et al. (7) found lower isolation yields in liver tissue from donors subjected to vascular clamping during resection surgery and therefore to ischemia, regardless of whether this had been continuous or intermittent. Furthermore, the ischemia time after a decrease in the number of viable cells obtained per gram of resected tissue was observed was set at 30 min. However, later, Lloyd et al. (8), evaluating the viability of hepatocytes isolated from liver resections, found no effect due to clamping of the hepatic artery and portal vein during hepatectomy, and Richert et al. (9) only found it when clamping was performed intermittently (7–40 min on, 5–15 min off), and not when continuous clamping was used. Thus, although the effect of vascular occlusion during hepatectomy on liver parenchyma is rather plausible, it is neither clear nor well characterized.

On the other hand, as mentioned above, factors derived from the donor’s characteristics of the resected liver tissue can also determine the number and quality of the isolated hepatocytes. Factors such as age, preoperative serum levels, the type of liver disease at the time of surgery, or chemotherapy treatment prior to hepatectomy are some of these donor-derived factors that different studies have pointed out to be related either to cellular isolation yield, viability, or even the functionality of the isolated hepatocytes. Nevertheless, the heterogeneity of the results found in the different studies does not allow conclusions to be drawn on the specific effect of each one of them (8, 10–12). For example, although different studies have shown that donor age can negatively influence isolation quality, there is still considerable controversy in this respect. While some authors have observed lower viability in hepatocytes isolated from liver tissue from resections carried out in patients older than 60 years (11), others have to raise this age to 80 to obtain a significant decrease in cell yield and viability (13). Even others do not find a significant effect of this donor factor on the yield of viable hepatocytes obtained from human liver resections (9).

Thus, the present work aims to determine the effect of both the characteristics of patients undergoing liver resection and vascular clamping during surgery on the yield, viability, and functional quality of primary human hepatocytes isolated from resected liver tissue.

## Materials and Methods

### Study Design

A cross-sectional observational prospective study was performed in which the cell isolation yield and the viability and functioning of hepatocytes isolated from hepatic tissue samples obtained from patients undergoing hepatic resection were analyzed.

### Patients and Surgery

All the hepatic tissue samples resected were obtained with prior informed consent and processed following the established norms after approval by the Aragon Research Ethics Committee.

All the samples were from patients older than 18 years of age; free of active infection; with negative serology for human immunodeficiency virus and hepatitis B and C; with bilirubin values in serum lower than 2 mg/dl, free of advanced liver disease (Child B-C cirrhosis) and macroscopic steatosis who underwent hepatic resection.

If vascular clamping was employed, the technique was total vascular exclusion with preservation of inferior vena cava flow, as this technique is considered safe, well-tolerated, and easily reproducible. For this purpose, double clamping was performed on both the hepatic pedicle with its arterial and portal branches using the Pringle maneuver and on the suprahepatic veins.

After each resection, the surgeon acquired a tissue sample resected from a section of macroscopically healthy hepatic tissue. Each sample was immediately placed in cold Wisconsin solution (ViaSpan, Bristol-Myers Squibb Pharma Ltd; Madrid; Spain) at 4°C. Within 45 min (a period documented in previous studies to be compatible with the isolation of large numbers of intact and viable human hepatocytes) (14), the tissue was transported to the Laboratory of Digestive Pathology at the Aragon Institute for Health Research, where the hepatocytes were isolated and *in vitro* functional tests were performed.

### Isolation of Human Hepatocytes

Hepatocyte isolation was carried out by the two-step collagenase perfusion technique described by Strom et al. (15), with modifications described by Solanas et al. (16). Briefly, once in the laboratory, each sample was weighed and perfused with two buffer solutions at 37°C: 300 ml of washing solution (Krebs-Ringer solution containing 0.5 mM EGTA and 1 nM nitro-L-arginine-methyl ester) followed by 100 ml of digestion solution (Krebs-Ringer solution with 5 mM CaCl2 and 0.05% collagenase). Both solutions were perfused at a speed of 40 ml/min/cannula, and the digestion solution was kept circulating until total tissue digestion was observed. The digested hepatic tissue was separated from the remaining connective tissue in a Krebs-Ringer solution kept on ice and subsequently filtered, first through four layers of sterile gauze and then through a 100-μm-pore filter. The solution containing isolated hepatocytes was centrifuged and washed three times. The resulting final pellet was suspended in a known volume of culture medium composed of Ham’s F-12/WEM (1:1, v/v) supplemented with 2% fetal bovine serum (FBS), 0.1% fraction V bovine serum albumin, 10 nM insulin, 25 μg/ml of transferrin, 26 mM sodium bicarbonate, 66.8 μM ethanolamine, 7.2 μM linoleic acid, 7 mM glucose, 0.62 mM ascorbic acid, 2 mM L-glutamine, 0.64 mM N-Nitro-L-Arginine methyl ester, 100 U/ml penicillin and 100 μg/ml amphotericin B. All chemicals were acquired from Sigma-Aldrich (St. Louis, MO, United States) except for WEM, Ham’s F-12 medium, FBS, and the antibiotic/antimycotic solution acquired from Lonza (Verviers, Belgium).

### Hepatocyte Viability, Cell Yield, and Function *in vitro*

Using an aliquot of cell solution resulting from isolation, the total number of cells obtained and cell viability was determined using the Trypan blue exclusion method. The cell isolation yield was calculated by dividing the number of viable isolated hepatocytes by grams of resected tissue.

For all functionality tests, cells were seeded on 96-well plates pre-coated with type I collagen (Biocoat, BD Bioscience, Belgium) at a density of 30,000 live cells/well, in 100 μl total culture medium/well, and were incubated at 37°C in an atmosphere of 5% CO2 and 95% air for 18 h.

Dehydrogenase activity was assessed by the MTT assay, based on the reduction of the tetrazolium salt, 3-(4,5-dimethylthiazol-2-yl)-2,5-diphenyltetrazolium bromide, into a purple formazan product. Albumin production was determined in 18-h supernatants using a Human Albumin ELISA Quantification Kit (Bethyl Laboratories, Inc., Montgomery, TX). The protein contents of adherent cells were measured using a Bicinchoninic Acid Protein Determination kit (Sigma-Aldrich).

### Variables Studied and Statistical Analysis

Patient-related parameters were collected, including age, gender, hepatic function prior to surgery (preoperative analytical values), liver disease, and whether there had been prior chemotherapy. In addition, surgery-related factors were also collected, including vascular clamping (whether performed or not, type of clamping, the total number of clampings performed, maximum time of each clamping, and total clamping time) were variables related to the isolation of hepatocytes from resected tissue and their subsequent culturing.

A Spearman bivariate analysis was performed to evaluate the linear relationships between continuous quantitative variables. The Chi-squared test was employed to show relationships between qualitative variables and was replaced by Fisher’s exact test when the application criteria were not met. The *T*-test or the non-parametric Mann-Whitney U test compared means between independent groups. The Kolmogorov-Smirnov test was used to analyze the normality of the variables. Finally, predictive models were constructed based on linear regression analysis to evaluate the influence of total time on the *in vitro* viability and yield of isolated hepatocytes. The level of statistical significance was established as a *p*-value less than 0.05. All analyses were performed with the SPSS software v15.0 for Windows (SPSS Ibérica, Madrid, Spain).

## Results

Isolation of hepatocytes was performed from liver tissue of 84 patients undergoing liver resection, although 8 of the isolations were unsuccessful due to technical problems during isolation. The characteristics of these patients before surgery and values of vascular occlusion maneuvers performed during liver resection are shown in Table 1. The mean age of patients was 61.4 years, ranging from 19 to 81 years. In a total of 70 patients (83.3%), the reason for surgery was a malignant disease, mainly colorectal cancer metastases (52.3% of cases). Of the patients with malignant pathology, 41 received chemotherapy treatment prior to resection surgery, with the average time from administration of neoadjuvant treatment to tissue resection being 6 ± 1.7 months, with a range of between 1 and 24 months. The mean analytical values of the patients before surgery were within the values expected for the healthy population, except for GGT values which were slightly higher. Data regarding surgical procedures were recorded in 67 of the 84 patients (Table 1). Of these patients, 43 suffered vascular occlusion during resection, 12 continuously and 31 intermittently. The mean total time of warm ischemia because of vascular exclusion during surgery was 36.8 ± 19.77 min.

**TABLE 1:** Patient characteristics prior to surgery and values of vascular occlusion maneuvers during liver resection.

Variables	Mean ± s.d.	Minimum	Maximum
Age (year)	61.4 ± 13.89	19	81
Gender, male (%, N)	59.5 (*N* = 50 of 84)		
Oncologic origin (%, N)	83.3 (*N* = 70 of 84)		
Chemotherapy previous to surgery (%, N)	58.6 (*N* = 41 of 70)		
Body mass index (BMI)	28.5 ± 4.53	17.3	45.6
Weight of the isolated liver tissue (g)	12.96 ± 0.929	2.01	31.52
**Preoperative liver function:**			
AST (U/L)	30.7 ± 19.72	11	119
ALT (U/L)	30.8 ± 29.79	8	202
GGT (U/L)	84.1 ± 89.30	13	500
AP (U/L)	123.1 ± 79.79	28	359
Albumin	3.9 ± 0.48	2.1	4.8
Bilirubin (mg/dL)	0.9 ± 0.64	0.31	4.3
Prothrombin act. (%)	101.1 ± 16.68	29	136
Hemoglobin (g/dL)	13.2 ± 1.83	8.6	17.1
Hematocrit (%)	39.2 ± 5.19	26.7	48.5
**Surgery-derived variables:**			
Vascular occlusion (%, N)	64.2 (*N* = 43 of 67)		
Continuous vascular occlusion (%, N)	27.9 (*N* = 12 of 43)		
Intermittent vascular occlusion (%, N)	72.1 (*N* = 31 of 43)		
Total time ischemia by vascular occlusion (min.)	36.8 ± 19.77	8	84

*s.d. = standard deviation; AST = aspartate aminotransferase; ALT = alanine aminotransferase; ALP = alkaline phosphatase; GGT = gamma-glutamyltransferase; AP = alkaline phosphatase; min. = minutes.*

### Effect of Patient-Donor Derived Factors

Table 2 shows the results obtained when analyzing variables collected from patients on the number, viability, and functionality of hepatocytes isolated from resected samples. The age and the BMI of the patients did not correlate (*p* > 0.05) with any of the variables under study, neither with the cell isolation yield nor with the viability and function of isolated hepatocytes, even when patients were divided into two age groups (group 1: <60 years, group 2: ≥60 years).

**TABLE 2:** Effect of patient- and surgical-associated factors on isolated hepatocytes’ number, viability, and function.

	Viability (%)		Cell yield (10^6^ cells/g liver tissue)		Cell adhesion (%)		MTT activity (absorbance)		Albumin (μ g/mg adhered protein)	
					
	Mean ± se/ *r*-value	*p-value*	Mean ± se/ *r*-value	*p-value*	Mean ± se/ *r*-value	*p-value*	Mean ± se/ *r*-value	*p-value*	Mean ± se/ *r*-value	*p-value*
Age	−0.201	0.082	−0.167	0.174	−0.274	0.105	−0.135	0.445	0.180	0.489
BMI	0.119	0.441	−0.120	0.472	−0.067	0.821	−0.096	0.680	−0.221	0.540
**Sex**										
Male	70.0 ± 1.28	0.018*	2.32 ± 0.267	0.035*	76.6 ± 2.43	0.174	0.768 ± 0.0707	0.023*	7.0 ± 1.23	0.333
Female	74.3 ± 1.07		4.51 ± 0.842		84.2 ± 4.67		0.435 ± 0.1008		4.0 ± 2.01	
**Surgery indication**										
Benign	76.6 ± 1.43	0.034*	5.46 ± 1.718	0.007*	87.7 ± 1.96	0.109	0.537 ± 0.1182	0.388	4.5 ± 3.4	0.535
Malign	70.9 ± 1.00		2.88 ± 0.366		76.8 ± 2.36		0.710 ± 0.0697		6.8 ± 1.17	
Chemotherapy										
No	73.5 ± 1.10	0.039*	3.94 ± 0.656	0.057*^t^*	78.1 ± 3.93	0.996	0.522 ± 0.0611	0.016*	4.7 ± 1.49	0.148
Yes	69.8 ± 1.41		2.37 ± 0.341		78.1 ± 2.37		0.821 ± 0.0850		7.9 ± 1.45	
**Vascular occlusion**										
No	74.6 ± 1.24	0.029*	4.14 ± 0.940	0.567*	79.5 ± 2.67	0.580	0.645 ± 0.0960	0.144	5.46 ± 0.955	0.933
Yes	70.4 ± 1.20		2.31 ± 0.282		77.6 ± 2.91		0.724 ± 0.0831		7.18 ± 1.641	
**Type of occlusion**										
Continuous	70.1 ± 3.04	0.788	2.72 ± 0.613	0.541	77.8 ± 3.65	1.000	0.396 ± 0.0800	0.006*	4.15 ± 0.450	1.000
Intermittent	70.5 ± 1.24		2.18 ± 0.318		77.5 ± 3.87		0.868 ± 0.0945		7.79 ± 1.924	
Vascular occlusion time	−0.332	0.010*	−0.309	0.024*	−0.178	0.395	0.288	0.105	0.265	0.274

se: standard error; r-value: Spearman correlation coefficient value; *statistically significant (p < 0.05); ^t^trend to signification.

**FIGURE 1:**
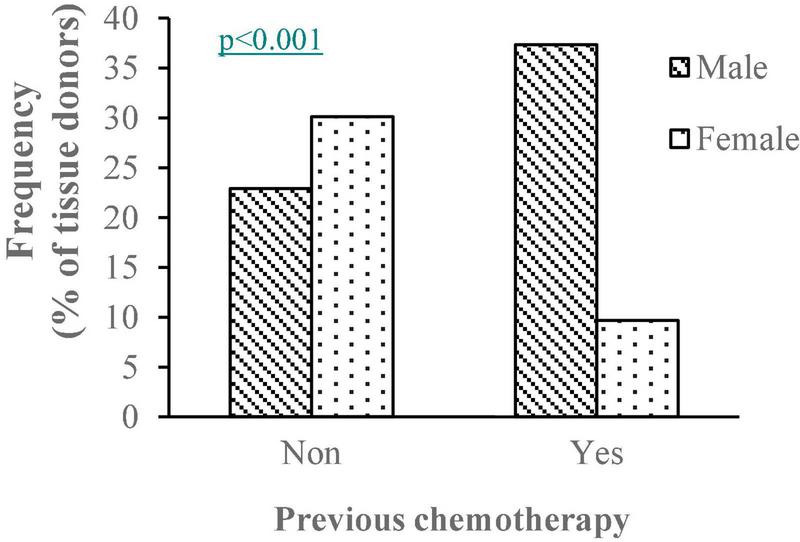
Percentage of males and females in samples with/without previous chemotherapy treatment. *^p^p*-value of the Chi-squared test between variables sex and previous chemotheapy treatment.

Hepatocyte viability was affected by the patient gender, being 6.3% higher in samples from women (*p* < 0.05), and by the type of tumor (malignant or benign) that motivated the surgery, resulting in higher viability when the reason was due to benign disease (76.6 ± 1.43% vs. 70.9 ± 1.00%, in benign and malignant disease, respectively, *p* > 0.05). Chemotherapy treatment prior to surgery also played a part, with greater hepatocyte viability in the absence of chemotherapy treatment (73.5 ± 1.10% without chemotherapy vs. 69.8 ± 1.41% with chemotherapy, *p* < 0.05). In addition, a significant association was found between the variables gender, indication for surgery and chemotherapy treatment (Chi-square = 11.4, *p* > 0.001 and Chi-square = 13.7, *p* > 0.001, respectively), with a higher percentage of previous chemotherapy treatment in men *versus* women and, as was logical, in patients undergoing surgery because of malignant disease *versus* benign disease (Figure 1).

Similarly, isolation cell yield was affected by the gender of the patients (*p* < 0.01), the malignancy of the disease motivating the surgery (*p* < 0.05), and the prior chemotherapy treatment (*p* = 0.058). Resection samples from interventions due to benign disease yielded almost twice the number of viable cells when compared with those from resections motivated by malignant disease (5.46 ± 1.718 vs. 2.88 ± 0.366 × 10^6^ cells/g liver tissue, *p* < 0.01). The yield in those samples that had not undergone prior chemotherapy treatment was 3.94 ± 0.656 cells/g liver tissue versus a yield of 2.37 ± 0.341 × 10^6^ cells/g liver tissue in samples with prior chemotherapy. The malignant disease also affected the adhesion of isolated hepatocytes when cultured, being higher in hepatocytes from cancer-free livers (87.7 ± 3.40 vs. 75.17 ± 10.58% adhesion in non-malignant vs. malignant resections), although this was not statistically significant (*p* = 0.057). These factors did not affect the other functional parameters studied (MTT and albumin production).

Cell metabolism measured as MTT was affected both by previous chemotherapy treatment and by the gender of the tissue donor. Thus, activity was higher with previous chemotherapy treatment and hepatocytes derived from male patients (Table 2). However, this higher activity did not translate into changes (*p* > 0.05) in hepatocyte adhesion to cell culture plates or albumin production.

As for serum analytical variables determined in the patient’s blood prior to surgery, none of them correlated with the viability of the isolated hepatocytes (*p* > 0.05), although the number of cells obtained per gram of isolated tissue correlated negatively with serum liver enzymes. Thus, those patients who presented worse values of liver function at the enzymatic level and therefore higher values of AST (*p* < 0.05), ALT (*p* < 0.05), GGT (*p* < 0.001) showed lower isolation cell yield, Spearman correlation coefficients being −0.225, −0.240, and −0.462, respectively. These variables were also significantly correlated (*p* < 0.05 for AST and GGT; *p* < 0.01 for ALT) with albumin production of isolated hepatocytes in culture. The remaining analytical variables prior to surgery did not affect (*p* > 0.05) the function of hepatocytes as it was determined in culture.

The weight of resected tissue that underwent perfusion for tissue isolation did not affect hepatocyte viability. However, it did affect the total number of cells obtained, with a negative correlation (*r*-value = −0.300, *p* > 0.05) so that larger samples yielded a lower number of viable hepatocytes per gram of tissue.

### Effect of Variables Derived From Surgery

Vascular clamping during surgery harmed both hepatocyte viability (*p* < 0.05) and isolation cell yield, although in the latter case, it did not reach statistical significance (*p* = 0.057). On the other hand, liver samples from non-clamped patients resulted in cell viability 4.3 percentage units higher than those from clamped patient samples (74.6 ± 1.24% without clamping vs. 70.4 ± 1.20% with clamping), as well as 78% more viable hepatocytes per gram of perfused tissue (Table 2).

Neither hepatocyte adhesion to cell culture plates, MTT function, nor albumin production of isolated hepatocytes in culture was affected (*p* > 0.05) by clamping during surgery.

While the type of vascular occlusion, continuous and intermittent, did not affect either hepatocyte viability or isolation cell yield (*p* > 0.05), total ischemia time to which the tissue was subjected during surgery did, there being a negative correlation between clamping time and both variables. As clamping time increased, both parameters decreased, with correlation coefficients of −0.332 (*p* < 0.01) and −0.309 (*p* < 0.05) for cell viability and yield, respectively. Likewise, we observed that patients subjected to a clamping time longer than 15 min presented a significant decrease in cell viability, while this did not occur with lower clamping times.

## Discussion

Obtaining the most significant possible number of viable and functional hepatocytes from resected tissue is determined by multiple factors associated with the isolation process and others linked to the patient, donor of the sample, and the surgical resection process itself. Although the factors derived from the donor and associated with the surgery have been considered in different studies, they have not been fully identified, and there is considerable controversy and confusion in this regard.

Our study demonstrates that both patient and surgical factors influence the isolation of human primary hepatocytes from resected liver tissue, either in isolation cell yields, hepatocyte viability, or function.

As for patient-derived factors, one of those that affected both isolation cell yield and the viability of the isolated hepatocytes was chemotherapy treatment prior to partial hepatectomy. Thus, a decrease in the number of isolated hepatocytes/g tissue (*p* = 0.059) and lower hepatocyte viability (*p* < 0.05) were observed when patients/donors had been previously treated with chemotherapy. Previous results in the literature are contradictory, although the study with the largest sample size obtains results similar to ours (6, 12). On the other hand, in our study, most patients who received chemotherapy prior to resection were cases of colorectal cancer metastases, in which more than one-third received a treatment containing oxaliplatin, a known hepatotoxic (17). In that sense, when we analyzed the effect of receiving previously to surgery hepatotoxic chemotherapy regimens or not, we found that samples from patients who had received chemotherapy regimens with known hepatotoxic effects (containing irinotecan and/or oxaliplatin and/or fluorouracil) rendered less viable hepatocytes per gram of isolated tissue, since this variable significantly affected both the hepatocyte viability (69.3 ± 1.79% vs 73.0 ± 0.94%, *p* = 0.024) and isolation cell yield (1.86 vs 3.96 × 106 cells/g tissue, *p* = 0.012). However, no significant differences were found in albumin production by cultured hepatocytes (*p* > 0.05) because of having received hepatotoxic chemotherapy regimens.

The negative effect of chemotherapy on hepatocyte isolation could also be related to the fact that samples from males and those obtained from resections due to malignant disease (mainly colorectal cancer metastases) yielded a lower number of hepatocytes and with lower viability since a significant association was found between gender, the indication of surgery and previous chemotherapy treatment (Figure 1).

Cell yield was also inversely related to pre-surgery transaminase values. These values could reflect previous liver injury either because of the chemotherapy or the tumor disease itself. In addition, hepatocytes from patients with elevated transaminases showed lower albumin production when cultured *in vitro* after isolation. This may reflect that these hepatocytes possess a lower capacity for protein synthesis, probably secondary to injury, and therefore they could not become fully functional for subsequent use.

The metabolic activity of the isolated cells (MTT activity) was higher for hepatocytes isolated from tissue with previous chemotherapy. This might explain, as in the case of viability, the higher activity found in hepatocytes from men. However, the differences in the metabolic activity of hepatocytes from men and women may also be due to the marked sexual dimorphism existing in the expression of metabolic genes in the liver (18).

We found no other correlations of any other patient-derived variable on the number and viability of isolated hepatocytes. According to our results, donor age did not significantly influence isolated hepatocytes’ number, viability, or function. Although some studies have shown that older age leads to a decrease in hepatocyte viability and isolation yields (8, 11, 14), our results are in agreement with others who found no such effects (9, 19, 20). These differences between studies could be due to the age range of tissue donors used, since when an age effect has been observed, substantial percentages of very young donors (less than 20 years old) (14) or donors over 80 years old have been included. This is explained by the greater viability of the isolated hepatocytes, which has been demonstrated in young donors (21), and the adverse effects on hepatocyte viability and yields on donors >80 years old, according to some studies (13). Moreover, when the effect of donor age on the viability of isolated hepatocytes has been observed, the correlation coefficient between both variables has been very low (8). Although statistically significant, there would not be a clear relationship at a biological level. Considering all of the above, we can conclude that donor age, within our age range, between 19 and 81 years, by itself, does not seem to influence the number and viability of isolated hepatocytes.

Our study shows that tissue warm ischemia significantly decreases hepatocyte viability and the number of viable cells per gram of isolated tissue when considering the resection procedures during surgery. In the latter case, the differences observed only reached a tendency to statistical significance (*p* = 0.057). At the functional level in culture, warm ischemia did not affect isolated hepatocytes, neither on MTT activity nor albumin production.

Clamping maneuvers are a constant variable in studies when identifying the best liver samples for hepatocyte isolation from resected tissue. Studies such as that of Alexandre et al. (7) or Lee et al. (10), the most numerous published to date, show results similar to those obtained in our series. In fact, in some studies that contradict these results, very few hepatectomies with vascular clamping are included, so they cannot be considered conclusive in this sense (22).

On the other hand, as in the few studies carried out to date that include functional tests when analyzing the influence of warm ischemia on hepatocyte isolation (23), our study also found no significant effect of vascular clamping during hepatectomy on the functionality of isolated hepatocytes in culture.

The review published by the Cochrane group in 2007 (24) considers intermittent occlusion of the portal triad as the technique with the most significant number of published evidence regarding the safety of its use and its effectiveness in the protection of surgical bleeding, thus considering it the technique of choice in clinical practice. Although our results show no significant differences in hepatocyte viability or isolation cell yield when comparing donor samples subjected to continuous or intermittent clamping, total ischemia times between patients undergoing continuous and intermittent clamping do not show homogeneous distribution. While continuous clamping never exceeded 30 min, total ischemia time varies from 20 to 84 min when intermittent clamping was carried out. So, variability in intermittent clamping duration could have made it more complex to obtain statistically significant results. As for the functional liver tests, significant differences were observed in the MTT parameter, obtaining higher values in the samples subjected to intermittent clamping than those subjected to continuous clamping. Thus, it could be considered a protective factor in the functional quality of human isolated hepatocytes.

Although the intermittent use of clamping during liver resection is recommended in clinical practice, literature shows contradictory results regarding its effect on hepatocytes’ number, viability, or function in the isolated cells from resected tissue. Findings obtained by Wagenssveld et al. (25) and Alexandre et al. (7) indicated that intermittent clamping was more detrimental to liver tissue than continuous clamping. However, in a later work, Richert et al. (9) comparing different reperfusion times within intermittent clamping, found a significant effect of this type of clamping on the number of viable cells only in those cases in which total ischemia times were longer. This suggests that cell damage does not appear to be related only to post-ischemic vasoconstriction caused by interruption of blood supply as suggested by Uhlmann et al. (26) and Wagensveld et al. (25), but also by ischemia time *per se*, as previously discussed by Lloyd et al. (8).

For a long time, it was feared that the warm ischemia produced during vascular clamping would cause cell damage and post-operative liver failure because of depriving the liver parenchyma of oxygen. This fact was studied in multiple studies that determined that healthy and cirrhotic livers could withstand up to 120 and 30 min of warm ischemia, respectively, without suffering significant alterations (27–29). The results of our study agree with these studies, so it was found a significant negative effect of the total vascular clamping time on the hepatocyte viability and isolation cell yield, although these effects were observed with ischemia times much lower than those recommended. Alexandre et al. (7) also described this negative correlation between ischemia extension time and the percentage of cell viability after isolation. They considered that patients subjected to a total ischemia time of more than 30 min presented a significant decrease in the viability of isolated hepatocytes. These results were subsequently corroborated in other studies (23). Therefore, this ischemia time due to vascular occlusion after which the hepatic parenchyma can be affected at the level of viability of isolated hepatocytes was reduced in our study to only 15 min so that it might be advisable to review the vascular clamping times during liver resection in clinical practice in order to minimize damage at the cellular level of the hepatic parenchyma.

Therefore, our results can conclude that previous chemotherapy treatment in liver tissue donors can influence the number, viability, and functionality of hepatocytes isolated from the resected tissue after partial hepatectomy. When starting from samples in which there is no advanced liver damage, such as in cirrhosis, steatosis, or with serum bilirubin levels higher than 2 mg/dl (exclusion criteria set in the study), it seems that factors such as donor age or body mass index do not influence hepatocyte isolation parameters, cell yield, viability or function. Vascular clamping during resection decreases both viability and the number of hepatocytes obtained, regardless of whether it is performed continuously or intermittently. The total clamping and, therefore, ischemia time are essential. According to previous studies, the warm ischemia time after which a decrease in isolated hepatocyte viability was observed was lower than that considered safe for the clinical practice of liver resection.

## Data Availability Statement

The raw data supporting the conclusions of this article will be made available by the authors, without undue reservation.

## Ethics Statement

The studies involving human participants were reviewed and approved by Comité de Ética de la Investigación de la Comunidad Autónoma de Aragón: CEICA. The patients/participants provided their written informed consent to participate in this study.

## Author Contributions

ES, MS, and AS conceived and designed the study. NS-F, LC, and AS were involved in patient care and sample and data collection. ES, NS-F, ALu, SL, and ALa performed laboratory determinations and data analysis and interpretation. ES, NS-F, PB, ALa, and MS drafted the manuscript. All authors revised and approved the final version of the manuscript.

## Conflict of Interest

The authors declare that the research was conducted in the absence of any commercial or financial relationships that could be construed as a potential conflict of interest.

## Publisher’s Note

All claims expressed in this article are solely those of the authors and do not necessarily represent those of their affiliated organizations, or those of the publisher, the editors and the reviewers. Any product that may be evaluated in this article, or claim that may be made by its manufacturer, is not guaranteed or endorsed by the publisher.
